# The Effectiveness of an Electronic Decision Support Algorithm to Optimize Recommendations of SGLT2i and GLP-1RA in Patients with Type 2 Diabetes upon Discharge from Internal Medicine Wards †

**DOI:** 10.3390/jcm14072170

**Published:** 2025-03-22

**Authors:** Irit Ayalon-Dangur, Emily Jaffe, Alon Grossman, Hagit Hendel, Yossi Oved, Amir Shaked, Ilan Shimon, Bar Basharim, Mohamad Abo Molhem, Rotem McNeil, Ran Abuhasira, Tal Shitrit, Limor Azulay Gitter, Reem El Saleh, Tzippy Shochat, Noa Eliakim-Raz

**Affiliations:** 1 Faculty of Medicine, Tel Aviv University, Tel Aviv 69978, Israel; 2 Department of Endocrinology, Rabin Medical Center, Petah Tikva 49414, Israel; 3 Internal Medicine B, Rabin Medical Center, Petah Tikva 49414, Israeltalshitrit123@gmail.com (T.S.); 4 Hospital Information Systems, Rabin Medical Center, Petah Tikva 49414, Israel; 5 Internal Medicine E, Rabin Medical Center, Petah Tikva 49414, Israel; 6 Internal Medicine A, Rabin Medical Center, Petah Tikva 49414, Israel; 7 Internal Medicine C, Rabin Medical Center, Petah Tikva 49414, Israel; 8 Internal Medicine D, Rabin Medical Center, Petah Tikva 49414, Israel; 9 Department of Biostatistics, Rabin Medical Center, Beilinson Campus, Petah Tikva 49414, Israel

**Keywords:** electronic decision support algorithm, clinical decision support system, SGLT2i, GLP-1RA, type 2 diabetes

## Abstract

**Background/Objectives:** Despite the established cardiovascular benefit of sodium–glucose cotransporter-2 inhibitors (SGLT2is) and glucagon-like peptide-1 receptor agonists (GLP-1RAs), these medications are under-prescribed in patients with type 2 diabetes. Our study aims to examine the effectiveness of a clinical decision support system (CDSS) in improving the recommendation rate of SGLT2i and GLP-1RA upon discharge. **Methods:** We developed an algorithm to automatically recommend SGLT2is and GLP-1RAs for eligible patients with type 2 diabetes upon discharge, based on current guidelines. Data were collected from electronic medical records of all eligible patients ≥18 years old hospitalized in one of five internal medicine wards at Beilinson Hospital. The primary outcome was to evaluate the rate of physician recommendation of SGLT2is and GLP-1RAs at discharge, before and after algorithm implementation. **Results:** Our study included 1318 patients in the pre-algorithm group and 970 in the post-algorithm group. The recommendation rate of SGLT2is and GLP-1RAs was 8.5% in the pre-algorithm group and 22.7% in the post-algorithm. The odds ratio (OR) of recommendation in the post- vs. pre-algorithm group was 3.151 (95% CI: 2.467–4.025, *p* < 0.0001). Recommendation rates increased in all subgroups analyzed, notably in patients hospitalized due to heart failure (recommendation rate pre-algorithm: 14.6% vs. post-algorithm: 49.02%). **Conclusions:** This study demonstrates the benefit of a CDSS in improving the recommendation rate of SGLT2is and GLP-1RAs in patients with type 2 diabetes upon discharge from hospitalization. Future studies should assess the impact of the algorithm on recommendation rates in other wards, medication utilization, and long-term outcomes.

## 1. Introduction

The prevalence of type 2 diabetes mellitus has risen significantly in the past decade and is a major cause of morbidity and mortality [1,2]. Patients with type 2 diabetes are at increased risk of microvascular complications of diabetes as well as atherosclerotic cardiovascular disease (ASCVD) and death from ASCVD compared to adults without diabetes [3]. Many studies have demonstrated that sodium–glucose cotransporter-2 inhibitors (SGLT2is) and glucagon-like peptide-1 receptor agonists (GLP-1RAs) significantly reduce major adverse cardiovascular events (MACE), the risk of worsening heart failure (HF), hospitalizations due to HF, arrhythmic events in patients with HF [4], and overall mortality in patients with type 2 diabetes [5,6,7]. They have also been shown to have beneficial effects in patients with chronic kidney disease (CKD) [6,7,8]. Contraindications of SGL2Tis include type 1 diabetes, end-stage renal disease, pregnancy, and breast feeding [9]. Contraindications of GLP-1RAs include end-stage renal disease, medullary thyroid cancer, MEN2, pregnancy and breast feeding, with increased caution in patients with a history of pancreatitis [9].

Current guidelines provided by the American College of Cardiology (ACC), American Diabetes Association (ADA), European Society of Cardiology (ESC), and European Association for the Study of Diabetes (EASD) recommend the use of GLP-1RAs or SGLT2is in patients with type 2 diabetes with or at high risk of cardiovascular disease (CVD), regardless of hemoglobin A1c (HbA1c) status or target and independently of metformin therapy [9,10,11,12]. In Israel, the Ministry of Health determines which medications are covered by the government, along with their specific approved uses for reimbursement, as outlined in the ‘Health Basket’. Medications included in the ‘Health Basket’ are available at a very low cost to all citizens, as Israel operates a national health insurance system that provides universal coverage. Physicians may prescribe medications according to their clinical judgement, but if a treatment is not included in the ‘Health Basket’, a patient may have to pay out of pocket. Indications for SGLT2is that are included in the ‘Health Basket’ are type 2 diabetes with HbA1c > 7.0%, HF, ASCVD, or CKD [13]. GLP-1RAs are included in the ‘Health Basket’ for use in patients with type 2 diabetes with HbA1c > 7.5% and BMI > 28 kg/m^2^ or for patients with BMI > 25 kg/m^2^ and CVD [13].

Despite the established cardiovascular benefit of these drugs, many studies have shown that these medications are under-prescribed in patients with type 2 diabetes and CVD [14,15,16,17]. A study conducted in the University of Mississippi Medical Center from January 2013 to June 2019 found that the percentage of patients with type 2 diabetes and CVD that were treated with SGLT2is and GLP-1RAs was as low as 1.4% and 1.6%, respectively [18]. There is extensive evidence of clinical inertia, defined as failure to initiate or intensify therapy when there is a true clinical indication, during inpatient management or discharge [19,20]. This clinical inertia can hinder proper management of patients with type 2 diabetes. Phillip H. Lee et al. showed that patients whose home diabetes regimen was intensified at discharge were less likely to be readmitted to the hospital compared with patients whose regimen was not intensified [21]. Thus, the adjustment of patients’ diabetes treatment regimen is crucial for proper management of these patients.

In a recently published study, Neha J. Pagidipati et al. showed that a coordinated, multifaceted intervention increased prescriptions of three groups of evidence-based therapies, including SGLT2is and GLP-1RAs, in adults with type 2 diabetes and ASCVD, highlighting the effectiveness of such interventions [22]. Another tool that can help increase the rates of prescription or physician recommendation of these medications is the implementation of computer clinical decision support systems (CDSSs). CDSSs can be knowledge-based, in which meaning is generated from algorithms that use current guidelines and information that is explicitly in a patient’s medical record, or non-knowledge-based, in which meaning is generated from machine learning and artificial intelligence [23]. In diabetes management specifically, many studies have demonstrated the important role of CDSSs in glycemic control with insulin [24]. Studies have also shown the positive impact of CDSSs that recommend various examinations, lab tests, and vaccines to improve outcomes of patients with diabetes [25,26]. The aim of the current study is to examine the effectiveness of an algorithm we developed as part of a knowledge-based CDSS to guide treatment decisions and assist in determining optimal recommendations for treatment with SGLT2is, GLP-1RAs, or both at the time of discharge from hospitalization. We hypothesize that the implementation of the algorithm will significantly improve the rate of recommendations of these medications in eligible patients at the time of hospital discharge.

## 2. Materials and Methods

### 2.1. Study Design, Setting, and Population

The study was conducted at Rabin Medical Center (RMC), Beilinson Hospital in Petah Tikva, Israel, a 900-bed primary and tertiary care, university-affiliated hospital. The study included patients ≥18 years old hospitalized for any reason, with a diagnosis of type 2 diabetes, who were eligible for SGLT2is and/or GLP-1RAs and were not already treated with these agents. Patients with type 1 diabetes were excluded. The data were collected from the electronic medical records (EMRs) of patients hospitalized in five out of the six internal medicine departments at Beilinson Hospital between 1 January 2021 and 31 December 2021 and between 1 April 2023 and 6 October 2023. Only the first hospitalization was included for patients that were hospitalized more than once during each period. The study was approved by the local institutional review board (RMC-0702-22, 7 December 2022).

### 2.2. The Algorithm

The algorithm was developed to recommend SGLT2is and/or GLP-1RAs for eligible patients with type 2 diabetes not already treated with these medications based on the current guidelines by the ACC, ADA, ESC, and EASD [9,10,11,12]. Although there are other approved indications for these medications, the algorithm in our study was only programmed to recommend these medications for patients with type 2 diabetes as the indication. Furthermore, the algorithm was programmed to recommend the medications according to eligibility approved for the ‘Health Basket’, except for glycated hemoglobin, which it disregards as long as the patient has diabetes. For SGLT2is, patients were eligible if they had type 2 diabetes and CKD, HF, or ASCVD. For GLP-1RAs, patients were eligible if they had type 2 diabetes and BMI > 28 or BMI > 25 with CVD. The algorithm works by gathering information about the patients from their EMRs, such as age, body mass index (BMI), glomerular filtration rate (GFR), comorbidities such as CVD and CKD, as well as established contraindications for therapy with GLP-1RAs and SGLT2is. Contraindications for GLP-1RAs that were included in the algorithm were a history of pancreatitis, pancreatic cancer, medullary thyroid cancer, or GFR < 15 mL/min/1.73 m^2^. The contraindication for SGLT2is was GFR < 30 mL/min/1.73 m^2^. A schematic presentation of the algorithm will be provided upon request. The algorithm was developed by internists and endocrinologists in collaboration with the computer unit in RMC in a programming language associated with the hospital’s EMR system, “Chameleon”. The algorithm was constructed during the period of January 2022–June 2022. Quality assurance by different researchers was conducted from June 2022 to October 2022 in order to assess the accuracy of the algorithm recommendations. The algorithm was implemented in the EMR system using a stepped-wedge trial design. One department adopted the algorithm in November 2022, followed by two departments in the second stage and the remaining wards in the third stage in April 2023. Once implemented, an algorithm button was present at the top of each patient’s file. The discharging physician could view the algorithm’s recommendation by clicking this button and then decide whether to incorporate the recommendation into the patient’s discharge letter.

### 2.3. Data Collection

Patients were divided into pre-algorithm (1 January 2021–31 December 2021) and post-algorithm (1 April 2023–6 October 2023) groups, corresponding to the algorithm’s implementation in April 2023. Data collection was prematurely stopped after 6 October 2023 as a result of the war in the region. The data collected included demographic details, length of stay (LOS), discharge diagnosis, comorbidities according to the Charlson comorbidity index (CCI) [27], and current antidiabetic medications. The discharge diagnosis in each patient was categorized as (1) type 2 diabetes complication (such as diabetic foot, hyperglycemia, or hypoglycemia), (2) HF, (3) ischemic heart disease (IHD), (4) ischemic stroke, (5) CKD, or (6) other (all other discharge diagnoses including pneumonia, urinary tract infection, gastroenteritis, delirium, observation before a procedure, etc.). The comorbidity of ASCVD was defined as IHD, cerebrovascular disease, or peripheral vascular disease. The comorbidity of type 2 diabetes complication includes diabetic nephropathy, diabetic neuropathy, and previous diabetic foot or amputation due to diabetic foot. The comorbidity of obesity was defined as a BMI greater than 30 kg/m^2^. Comorbidities were extracted from patient EMRs automatically using ICD diagnosis codes and free text. Demographic details, LOS, and medications were automatically collected as well. In addition, the algorithm’s recommendations were automatically recorded and the physicians’ recommendations in the discharge note were manually extracted.

### 2.4. Study Outcomes

The primary outcome we evaluated was the recommendation rate of GLP-1RAs and SGLT2is for eligible patients not already treated with these medications at the time of discharge from hospitalization in an internal medicine ward. We compared recommendation rates before algorithm implementation (1 January 2021–31 December 2021) to recommendation rates after algorithm implementation (1 April 2023–6 October 2023). The secondary outcome was the recommendation rates in various subgroups based on age, sex, comorbidities, current medications, and discharge diagnosis.

### 2.5. Statistical Analysis

The statistical analysis for this paper was generated using SAS Software, Version 9.4. Continuous variables were presented by the mean ± standard deviation or the median and interquartile range (IQR), and categorical variables were presented by (N,%). Normality of continuous variables was assessed using the Kolmogorov–Smirnov test. The *T*-Test was used to compare the value of normally distributed continuous variables between study groups, Wilcoxon was used for non-normal continuous variables, and Fisher’s exact test or the Chi-square test was used to compare the value of categorical variables between study groups. Univariate logistic regression was used to calculate odds ratios (ORs). Two-sided *p* values less than 0.05 were considered statistically significant.

## 3. Results

### 3.1. Patient Characteristics

The total number of patients with type 2 diabetes that were hospitalized in an internal medicine ward was 2735 and 1416 in the pre-algorithm and post-algorithm periods, respectively. Of them, a total of 1318 patients were included in the pre-algorithm group, and 970 were included in the post-algorithm group. All patients for whom the algorithm recommended these medications were included in the study, while those who did not receive a recommendation were excluded. The excluded population included those already on SGLT2is/GLP-1RAs, those who did not meet the eligibility criteria for these medications, those who had contraindications to these medications, or patients with type 1 diabetes. Thus, the patients that were included in this study were only those for whom the algorithm recommended at least one of these medications. Patient baseline characteristics are shown in Table 1. Mean age was 74.2 ± 11.7 years in the pre-algorithm group and 71.3 ± 12.5 years in the post-algorithm group (*p* < 0.0001).

The baseline characteristics of the cohorts before and after algorithm implementation are shown. Age and length of stay significantly differed between the two groups. The comorbidities of ASCVD, heart failure, and hypertension were significantly higher in the pre-algorithm group, whereas obesity and liver disease were significantly higher in the post-algorithm group. Patients in the pre-algorithm group were more likely to be taking at least one type 2 diabetes medication. The pre-algorithm cohort had significantly more patients with a discharge diagnosis of ischemic heart disease, whereas the post-algorithm cohort had significantly more patients with a discharge diagnosis of “other”, which included pneumonia, UTIs, pre- or post-operative observation, etc.

### 3.2. Overall Rate of Recommendation

**Figure 1:**
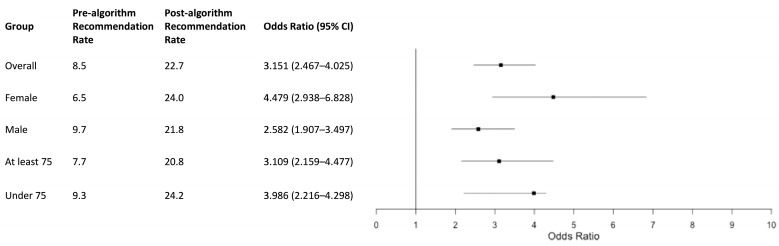
The rates of recommendation before and after algorithm implementation and the odds ratio of recommendation in the post- versus pre-algorithm groups for the overall cohort and by sex and age. The rate of recommendation improved in all groups, with an odds ratio and lower limit of the confidence interval greater than 1 in all groups.

The overall rate of recommendation of SGLT2is and GLP-1RAs for eligible patients increased from 8.5% in the pre-algorithm group to 22.7% in the post-algorithm group (Figure 1). The odds ratio (OR) of recommendation in the post- vs. pre-algorithm group was 3.151 (95% CI: 2.467–4.025, *p* < 0.0001) (Figure 1). The overall rate of recommendation in one internal medicine ward in our hospital that did not participate in the study was 5.4% during the pre-algorithm period and 6.9% during the post-algorithm period.

### 3.3. Analysis by Age and Sex

Patients under 75 years old had higher recommendation rates in both groups (pre-algorithm: 9.3%; post-algorithm: 24.2%) compared to those 75 years or older (pre-algorithm: 7.7%; post-algorithm: 20.8%) (Figure 1). However, the rates increased post-algorithm for both age groups. The OR of recommendation post- vs. pre-algorithm was 3.986 (95% CI: 2.216–4.298, *p* < 0.0001) in patients under 75 and 3.109 (95% CI: 2.159–4.477, *p* < 0.0001) in patients 75 and older (Figure 1). Both male and female patients showed increased recommendation rates post-algorithm, with a more pronounced improvement in females (Figure 1).

### 3.4. Analysis by Comorbidity

**Figure 2:**
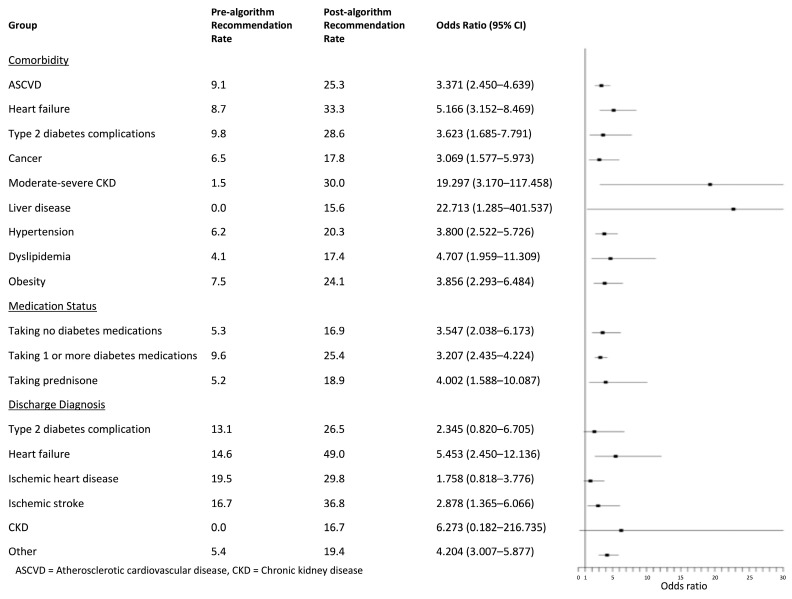
The rates of recommendation before and after algorithm implementation and the odds ratio of recommendation in the post- versus pre-algorithm groups by comorbidity, medication status, and discharge diagnosis. The rate of recommendation improved in all groups, with an odds ratio and lower limit of the confidence interval greater than 1 in all comorbidity and medication groups. The highest rate of recommendation was seen in patients with a comorbidity of heart failure after algorithm implementation, with a recommendation rate of 49.0%. The odds ratio and lower limit of the confidence interval was greater than 1 in patients with a discharge diagnosis of heart failure, ischemic stroke, and “other”.

The lowest rates of recommendation based on comorbidity were seen in patients with liver disease (pre-algorithm: 0%; post-algorithm: 15.6%) and cancer (pre-algorithm: 6.5%; post-algorithm: 17.8%) (Figure 2). In the post-algorithm group, the highest rates of recommendation were seen in patients with cardiovascular disease (25.3%), type 2 diabetes complications (28.6%), and HF (33.3%) (Figure 2). The rates of recommendation improved after algorithm implementation for all comorbidity subgroups that were assessed (Figure 2). The OR of recommendation post- vs. pre-algorithm was largest in patients with dyslipidemia (OR: 4.707, 95% CI: 1.959–11.309, *p* = 0.0005), HF (OR: 5.166, 95% CI: 3.152–8.469, *p* < 0.0001), moderate–severe CKD (OR: 19.297, 95% CI: 3.17–117.458, *p* = 0.0013), and liver disease (OR: 22.713, 95% CI: 1.285–401.537, *p* = 0.0331) (Figure 2).

### 3.5. Analysis by Medication Status

Patients taking no diabetes medications had lower rates of recommendation in both groups (pre-algorithm: 5.4%; post-algorithm: 17.0%) compared to those regularly taking at least one diabetes medication (pre-algorithm: 9.6%; post-algorithm: 25.4%) (Figure 2). The OR of recommendation post- vs. pre-algorithm was 3.547 (95% CI: 2.023–6.173, *p* < 0.0001) in patients taking no diabetes medications and 3.207 (95% CI: 2.435–4.224, *p* < 0.0001) in patients taking at least one diabetes medication (Figure 2). Patients taking prednisone regularly had lower recommendation rates in both the pre- and post-algorithm groups (pre-algorithm: 5.2%; post-algorithm: 19.0%) compared to patients not taking prednisone (pre-algorithm: 8.8%, post-algorithm: 23.2%) (Figure 2). However, the rates increased post-algorithm for both prednisone status groups, as the OR of recommendation post- vs. pre-algorithm was 4.002 (95% CI: 1.588–10.087, *p* = 0.0033) in patients taking prednisone and 3.113 (95% CI: 2.414–4.014, *p* < 0.0001) in patients not taking prednisone (Figure 2).

### 3.6. Analysis by Discharge Diagnosis

The highest rates of recommendation were observed in patients with a discharge diagnosis of HF (pre-algorithm: 14.61%; post-algorithm: 49.0%) and ischemic stroke (pre-algorithm: 16.7%; post-algorithm: 36.8%) (Figure 2). The lowest rates of recommendation were seen in patients with a discharge diagnosis of CKD (pre-algorithm: 0%; post-algorithm: 16.7%) and “other” (pre-algorithm: 5.4%; post-algorithm: 19.4%) (Figure 2). The OR of recommendation post vs. pre-algorithm was significant in patients with a discharge diagnosis of HF (OR: 5.453, 95% CI: 2.45–12.136, *p* < 0.0001) and ischemic stroke (OR: 2.878, 95% CI: 1.365–6.066, *p* = 0.0055) (Figure 2).

## 4. Discussion

The implementation of the algorithm was associated with a significant increase in the rate of recommendation of SGLT2is and GLP-1RAs, with an OR of recommendation post- vs. pre-algorithm of 3.151 in the overall cohort, indicating a significant positive impact of the algorithm on medication recommendation rates. Additionally, the recommendation rates increased across all analyzed subgroups. Our findings therefore support the use of a CDSS to enhance the appropriate treatment of patients with type 2 diabetes. Our results are consistent with previous studies demonstrating that CDSS implementation improves physician adherence to diabetes management guidelines [28] and is both effective and safe in improving management of patients with diabetes [29]. Additionally, Zhang et al. demonstrated that more than 80% of physicians believe that the use of a CDSS can help improve type 2 diabetes patient outcomes, suggesting strong physician support for CDSS implementation [30]. Moreover, knowledge-based algorithms like ours can be created for new medications and implemented according to current and updated guidelines, offering a cost-effective and lower-risk alternative compared to a non-knowledge-based CDSS. This makes it a feasible option for implementation in various clinical settings, especially for physicians with limited experience and access to medical resources, thereby potentially improving the management of type 2 diabetes on a broader scale.

Our study also provides insight into physician prescribing behaviors. The higher rate of recommendation in patients under 75 years old compared to those 75 and older was consistent with previous studies that observed lower prescription rates of these medications in older populations [14,31,32]. Addressing this disparity is crucial, as Diallo et al. found that a greater reduction in MACE is seen in elderly patients taking GLP-1RAs compared to younger patients [33]. The hesitation to prescribe these drugs is understandable given the advanced age of the patients, but their safety and efficacy in elderly individuals have been well documented [34]. That said, it was encouraging to see improvement in both age groups after algorithm implementation.

The lower rates of recommendation seen in patients with cancer were anticipated, as physicians are likely to be cautious about prescribing new regular medications for patients with a poor prognosis or those who are seriously ill and less able to manage new medications and their side effects. In contrast, higher recommendation rates seen in patients with ASCVD and HF were consistent with previous studies [14,20,31]. It is well established that ASCVD and HF are both indications for treatment with SGLT2is and/or GLP-1RAs, and thus the higher rates of recommendation seen in these patients were expected. Furthermore, the large improvement in the recommendation rate and OR seen in patients with HF as a comorbidity likely reflects the growing evidence and emphasis within the medical community on using these medications to reduce mortality and hospitalizations in patients with HF [5,6]. One study found that rates of SGLT2i and GLP-1RA utilization were lower in patients with HF before 2017, with trend reversal after 2017, likely due to the outpouring of evidence supporting the benefits of these medications [35].

The improved recommendation rate post-algorithm in patients with moderate–severe CKD might be explained by the growing evidence of renal benefits in using these medications [6,7]. Although the low rate of recommendation of 16.7% and a nonsignificant pre- vs. post-algorithm OR of recommendation was seen in patients with a discharge diagnosis of CKD, the number of patients with this discharge diagnosis was only six, and thus additional monitoring of the recommendation rates in these patients would provide a more accurate understanding of the impact of CKD on recommendation rate. It is also reasonable that patients discharging with worsening renal failure are less likely to start new medications that impact kidney function.

It is also important to note the possible impact of the Israeli medical system on recommendation rates of SGLT2i/GLP-1RA. SGLT2is have been included in the ‘Health Basket’ since January 2017, initially just for treatment of patients with type 2 diabetes. Since February 2022, HF and CKD were included as indications for SGLT2is, with different approval timelines for empagliflozin and dapagliflozin [13]. GLP-1RAs have been included in the ‘Health Basket’ since January 2010 for patients with type 2 diabetes [13]. Thus, it is likely that the change in coverage for these medications had an impact on the recommendation rate. Furthermore, the algorithm’s recommendations are based on the reimbursement policy for medications included in the ‘Health Basket’, except for glycated hemoglobin, which it disregards as long as the patient has diabetes. According to the ‘Health Basket’, SGLT2is are covered for patients with an HbA1c level above 7.0%, and GLP-1RAs for those with an HbA1c above 7.5%. However, the algorithm follows a similar approach to the ADA guidelines, which state that if there is an indication for SGLT2i or GLP-1RA treatment, they should be provided regardless of glycated hemoglobin levels. The algorithm may therefore suggest a medication that is not reimbursed, but since patients can benefit from its use and many private insurance options exist, it was still programmed to recommend the appropriate medications.

Patients taking no diabetes medications had lower rates of recommendation in both pre- and post-algorithm groups compared to patients taking at least one diabetes medication. This might be reflective of physicians’ reluctance to recommend these newer medications prior to initiating more common medications with extensive therapeutical experience such as metformin. However, the benefits of these medications are beyond glycemic control, and they should be considered when initiating medical therapy for type 2 diabetes irrespective of metformin treatment status.

The most pronounced effect of the algorithm was seen in patients with a discharge diagnosis of HF, who had the highest rate of recommendation of nearly 50% post-algorithm implementation. Conversely, patients with the lowest rates of recommendation and OR based on discharge diagnosis were those with a discharge diagnosis categorized as “other”. This suggests that when patients are hospitalized for reasons unrelated to diabetes or metabolic disease, such as an acute infection, physicians may be less focused on addressing chronic aspects of their patients’ health. The impact of discharge diagnosis on recommendation rates is logical, as the discharge diagnosis is foremost in the physician’s mind at the time of discharge, influencing their recommendations in their discharge letters. These findings highlight the role of context in clinical decision-making and suggest that more initiatives may be needed to ensure that chronic conditions like diabetes are consistently addressed, even when they are not the primary reason for hospitalization.

While physician discharge letter recommendation after hospitalization is only one part of the continuum of patient care, we believe that it is important to investigate the impact of the algorithm on this key step, as it has been shown to be a pivotal moment in re-evaluating patient management. Studies have shown that planning diabetes discharge interventions can have an enormous impact on patients’ glycemic control and readmissions [36,37]. A focused review published in 2019 found that team-based, patient-centered structured discharge planning per guideline recommendations can help improve transitions in care for patients with diabetes and improve patient outcomes by reducing medication errors, delay of care, and hospital readmissions [38]. Umpierrez et al. conducted an exploratory study to test the safety and efficacy of a designed algorithm for selecting discharge diabetes medications based on admission HbA1c [39]. After 12 weeks, HbA1c was reduced by 0.1% in patients with a baseline HbA1c of 7–9% and by 3.2% when baseline HbA1c > 9% [39]. The results of this study complement our study’s emphasis on the importance of reassessing the management of diabetic patients at discharge and the role of an algorithm to do so.

The algorithm was developed to assist physicians, addressing a clear need given the large number of diabetes patients and the limited number of experts. As the medical field becomes more technology-driven, it is essential to adapt to and work with these tools, despite the significant challenge of influencing physicians’ decision-making processes. It is crucial to note that the algorithm offered recommendations, but the final decision rested with the treating physician who signed the discharge letter and chose whether or not to follow the algorithm’s suggestions. Our aim was partly to remind the physicians about these medications when treating patients with type 2 diabetes, but the algorithm was not meant to replace the physicians’ discretion. The discrepancies between the algorithm’s and the physicians’ recommendations therefore may arise because some patients meet the algorithm’s criteria for treatment, yet physicians may decide against it due to factors the algorithm cannot account for when making its recommendation. These factors include the patient’s overall health status, frailty, cognitive ability, extensive metastatic disease, a history of low compliance, a history of significant weight loss, and more. Furthermore, physicians may have concerns about intensifying a patient’s regimen at discharge due to lack of confidence in new oral therapies, insufficient post-hospital surveillance, or a patient’s financial and social barriers. It is also important to note that the recommendation rate represents an average across the five wards, with compliance with the algorithm’s recommendation varying by ward. Each department naturally prioritizes different concerns, which results in differences in compliance with the algorithm’s recommendations.

Our study has several limitations. First, data collection ended prematurely due to the war in the region that began in October 2023. Second, we were limited by factors that might impact physicians’ recommendations such as physician experience, patient preference, or other clinical characteristics of patients, as discussed above, that were not able to be included in our knowledge-based algorithm. Furthermore, there is likely an effect of time and prescription trends on recommendation rate, as recommendations of these medications may changes as they are further studied and physicians become more comfortable with using them. Studies have shown an increase in recommendation or prescription rate of these medications over time; however, the rates of increase are not as major as the increase observed during our study period [31,40,41]. Importantly, the rate of recommendation in one internal medicine ward in our hospital that did not participate in the study was 5.4% during the pre-algorithm period and 6.9% during the post-algorithm period. This one ward does not act as a control group because it is only one ward and is therefore likely subject to biases of specific department behavior, but the minimal improvement in this ward’s recommendation rate during the same time period as our study provides further evidence that the algorithm was likely at least partially responsible for the increase in recommendation rate in the wards that did participate in the study. Another limitation is that due to the experimental nature of the algorithm, hospital policy required that physicians manually access the algorithm’s recommendation by clicking on it, rather than the algorithm’s recommendation automatically appearing on the screen. We believe that the effectiveness of the algorithm would have been even greater if the algorithm’s recommendations were automatically displayed. If this algorithm is able to be completely integrated into the hospital’s EMR system, we believe that the increase in physician recommendations is likely to be both greater and more sustainable over time. The last limitation is that data collection from both groups was carried out through retrospective EMR data extraction and there is a risk of misclassification bias. However, the presence or absence of the physicians’ recommendations in the discharge letter was manually verified.

In the future, studies assessing the recommendation rate of specific SGLT2is and GLP-1RAs would be beneficial in understanding the role of CDSSs in their utilization, as specific SGLT2i and GLP-1RA medications have been shown to have slightly different recommendation and prescription rates [31,41]. We did not assess the rates of recommendation of specific SGLT2i and GLP-1RA medications in our current study because most physicians recommended a drug class and not a specific medication in their discharge letter. Additionally, our study was conducted in internal medicine wards in one hospital, but the effect of the algorithm on rates of recommendation in other hospitals, healthcare systems, and clinical settings such as coronary care units, surgery wards, and primary care clinics is an important factor to be assessed in the future that would also improve generalizability. Lastly, our study analyzed the rate of physician recommendations in hospital discharge letters, which is only one step in the continuum of patient care. Therefore, future studies assessing the sustainability of the algorithm over time, the impact of the algorithm on physician prescription post-discharge, patient utilization of these medications, and patient outcomes are also essential to best understand the role and effectiveness of this CDSS in type 2 diabetes management.

## 5. Conclusions

This study demonstrates the benefit of a CDSS to improve the management of patients with type 2 diabetes by increasing recommendation rates of GLP-1RAs and SGLT2is upon discharge from hospitalization in an internal medicine ward. More complex and innovative systems can be developed in the future to account for more patient characteristics.

## Figures and Tables

**Table 1:** Baseline characteristics of patients in pre- and post-algorithm cohorts.

		Pre-Algorithm (N = 1318)	Post-Algorithm (N = 970)	*p* Value
Age (years)	Mean ± SD	74.2 ± 11.7	71.3 ± 12.5	<0.0001
Sex	Male	61.5%	60.4%	0.603
	Female	38.5%	39.6%	
LOS (days)	Median (IQR)	3 (2–6)	4 (3–7)	<0.0001
Comorbidity	ASCVD	60.8%	47.2%	<0.001
	Heart failure	25.2%	17.6%	<0.001
	Type 2 diabetes complications	10.1%	7.2%	0.017
	Cancer	18.7%	13.9%	0.003
	Obesity	20.3%	32.5%	<0.001
	Moderate–severe CKD	5.0%	3.1%	0.026
	Liver disease	4.5%	7.9%	<0.001
	Hypertension	47.3%	38.0%	<0.001
	Dyslipidemia	13.0%	11.9%	0.443
Type 2 Diabetes Medications	Taking one or more	74.4%	67.7%	<0.001
	Taking none	25.6%	32.3%	
Prednisone		8.7%	11.4%	0.033
Discharge Diagnosis	Type 2 diabetes complication	4.6%	3.5%	0.204
	Heart failure	6.8%	5.3%	0.158
	Ischemic heart disease	9.3%	4.8%	<0.001
	Ischemic stroke	7.7%	5.9%	0.096
	CKD	0.8%	0.6%	0.629
	Other	70.7%	79.9%	<0.001

## Data Availability

The original contributions presented in the study are included in the article, further inquiries can be directed to the corresponding authors.

## Abbreviations

SD = standard deviation; LOS = length of stay; IQR = interquartile range; ASCVD = atherosclerotic cardiovascular disease; CKD = chronic kidney disease; SGLT2i = sodium–glucose cotransporter-2 inhibitor; GLP-1RA = glucagon-like peptide-1 receptor agonist.
